# The Relationship between Visual-Spatial and Auditory-Verbal Working Memory Span in Senegalese and Ugandan Children

**DOI:** 10.1371/journal.pone.0008914

**Published:** 2010-01-27

**Authors:** Michael J. Boivin, Paul Bangirana, Rebecca C. Smith

**Affiliations:** 1 International Psychiatric and Epidemiologic Program, Michigan State University, East Lansing, Michigan, United States of America; 2 Neuropsychology Section, Department of Psychiatry, University of Michigan, Ann Arbor, Michigan, United States of America; 3 Department of Psychiatry, Makerere University School of Medicine, Kampala, Uganda; 4 Graduate Psychology Program, Chestnut Hills Professional School of Psychology, Philadelphia, Pennsylvania, United States of America; University of Granada, Spain

## Abstract

**Background:**

Using the Kaufman Assessment Battery for Children (K-ABC) Conant et al. (1999) observed that visual and auditory working memory (WM) span were independent in both younger and older children from DR Congo, but related in older American children and in Lao children [1]. The present study evaluated whether visual and auditory WM span were independent in Ugandan and Senegalese children.

**Method:**

In a linear regression analysis we used visual (Spatial Memory, Hand Movements) and auditory (Number Recall) WM along with education and physical development (weight/height) as predictors. The predicted variable in this analysis was Word Order, which is a verbal memory task that has both visual and auditory memory components.

**Results:**

Both the younger (<8.5 yrs) and older (>8.5 yrs) Ugandan children had auditory memory span (Number Recall) that was strongly predictive of Word Order performance. For both the younger and older groups of Senegalese children, only visual WM span (Spatial Memory) was strongly predictive of Word Order. Number Recall was not significantly predictive of Word Order in either age group.

**Conclusions:**

It is possible that greater literacy from more schooling for the Ugandan age groups mediated their greater degree of interdependence between auditory and verbal WM. Our findings support those of Conant et al., who observed in their cross-cultural comparisons that stronger education seemed to enhance the dominance of the phonological-auditory processing loop for WM.

## Introduction

In what has become the dominant model of memory organization, Baddeley and Hitch postulated a distinction between the control processes in memory and the structural memory stores [2]. At the heart of the control processes is what is commonly referred to as “working memory”, which is comprised of two modality-specific subsidiary systems that serve an attention-based central or executive process. The first of the working memory systems is the visual-spatial sketch pad. This system is involved in the processing and memory of information of a more distinctively visual and/or spatial nature and is often instrumental in more adaptive motor control and response. The other subsidiary system is the phonological loop, which is involved in the processing and memory of auditory verbal material.

Gathercole and colleagues proposed that the central executive, phonological loop, and the visual-spatial sketchpad were all in place by age six, and that the two subservient systems have a stronger relationship with the central executive than with each other. After that age, there is an absence of any type of major developmental changes in the strength of the relationship between the three structures [3].

Others have proposed that there are major developmental shifts in the relationship between visual-spatial and phonological-verbal WM as children develop. Working memory (WM) in early childhood tends to be characterized by strategies consistent with the use of the visual-spatial sketch pad. Storage and retrieval of remembered information tends to also be more dependent on visual-spatial cues. An example of this is when preschoolers and early-elementary school children rely primarily on visual inspection of items to be remembered, which might be accompanied by such motor responses as pointing at the items [4], [5]. However, as children get older, particularly beyond the ages of eight or nine years, working memory tends to depend more on phonetic or semantically-based verbal strategies for remembering information. This may involve a greater dependence on verbal description, rehearsal, or categorization in order to remember information, even if those items are distinguished by visual-spatial or motor-based features [6].

Using various memory subtests from the Kaufman Assessment Battery for Children (K-ABC) [7], Conant and colleagues evaluated the correlation between visual-spatial and phonological-auditory memory span (MS) in younger (5 through 8 yrs) and older (9 to 12 yrs) American children and children from DR Congo. A lack of an association between the two types of working memory would indicate that they operated independently of one another.

Conant hypothesized that in younger children, the two memory systems would be independent. However, as children were educated and become literate, the auditory-phonological system would dominate WM through verbal mediation. Consequently, the two memory systems would become more strongly associated. Conant's hypothesis was supported by her findings in younger American children, where visual and auditory WM span were not strongly associated. However, they were strongly associated in older American children.

For children from DR Congo, the younger age group had weak associations between visual and auditory WM span. In contrast to the American children, the older age group did not show the shift from visual to verbal as evidenced by a strong association in WM span [8]. Conant concluded that this difference could be due the lack of formal education among the Congolese children in their rural setting, as well as the emphasis on oral language instead of written. However, there was also the possibility that the differences resulted from the economically poor living conditions characterizing their rural developmental milieu.

Using the same K-ABC subtests, Conant and colleagues then compared children from America and Laos [1]. While the younger children of both cultural groups had weak associations between visual and auditory WM span, both cultural groups of older children had strong associations, demonstrating the shift in working memory. They concluded that like American children, the Lao children, many of whom were from a more affluent urban setting, had a strong and effective literacy component within their educational system. Consequently, the auditory-verbal WM dominance shift was due to literacy training, rather than a universal cognitive developmental change. This would support Vygotsky's theory that language is the foundation of the cognitive schemas that are prominent within a given cultural context [9].

In a previous study with these Senegalese children, Boivin observed that children from rural settings had a greater likelihood of having a history of recurring malaria, more severe intestinal parasite infections, and lower K-ABC global cognitive ability scores [10]. Quality of home environment and more education in Ugandan children were predictive of K-ABC WM performance; while nutritional well-being was predictive of visual spatial processing, spatial memory, and attention [11]. We hypothesized that in our present cross-cultural comparison, better education and nutritional well-being would facilitate a greater association between visual-spatial and auditory-phonological WM. Subsequently, the auditory-phonological system would become dominant in verbal memory.

## Methods

### Participants

#### Children from Senegal

Appropriate IRB approval was obtained through Indiana Wesleyan University (MJB) and through the West African Research Center (WARC) in Dakar, Senegal. Informed consent was obtained in writing from the mother or principal caregiver of each study child. Thirty-five female and 41 male school-age children ranging from 6 to 15 years of age comprised the K-ABC sample for this cultural group. Of these children, 50 were from village areas and 26 were from an urban setting. The rural Senegalese children were recruited from two locations and evaluated in those settings. Thirty of the children were recruited in the village of Maka Saar, a rural area about 100 km north of Dakar. Twenty children were recruited from the village of Bandia, a rural area about 30 km west of Dakar. The remaining children were urban children recruited in Dakar at an outpatient clinic (n = 12) and through a local primary school in Fann, Dakar (n = 14), which is a middle-class area of the city. The children from Bandia and Dakar were healthy controls used as a comparison group in a study evaluating the effects of a history of cerebral malaria on cognitive performance [10].

#### Children from Uganda

Appropriate IRB approvals were obtained through Indiana Wesleyan University (MJB), Case Western University, and Makerere University in Kampala, Uganda. Informed consent was obtained in writing from the mother or principal caregiver of each study child. The urban sample for this culture was comprised of community controls (53 females, 49 males) recruited for two studies that took place in Kampala, Uganda [12], [13]. We also included 30 children in the 1^st^ grade at an elite private school in Kampala, and 79 control children from Kampala who served as a control group in a neuropsychological study of the effects of HIV [14]. No children were included with a history of cerebral malaria or other known CNS disease requiring hospitalization. Neither were children included who were developmentally at risk because they had lost a principal caregiver to HIV.

### K-ABC Assessment

Age-appropriate subscales for the Sequential and Simultaneous Processing portion of the Kaufman Assessment Battery for Children (K-ABC, 1^st^ edition) were administered to children in their dominant local language by native speakers. Age was verified from the medical passbook (medical record book) for the child which is typically in the possession of the parent or principal caregiver. The present analysis included spatial memory (visual-spatial memory) as well as the three subtests used by Conant et al. (1999, 2003) in their studies. These were the Sequential Processing subtests of Hand Movements (visual memory span), Number Recall (auditory memory span), and Word Order (both visual and auditory). The K-ABC subtests used in the present analysis are described below.

#### Hand Movements (HM)

This is a visual-kinesthetic memory-span task in which the examiner taps on a table with the fist, palm, or side of the hand and the participant repeats the tap sequence. The hand movements vary in order and are progressively longer with each successive subtest item. This task is considered to be a measure of visual WM span because the child sees the progressively longer tapping sequence, and then repeats the same sequence of motor hand movements.

#### Spatial Memory (SM)

Colorful nonsense objects within a matrix array are presented to a child for 5 seconds. A blank matrix is then presented and the child is asked to point to the cells that formerly contained the various objects. More objects are presented during the 5 sec exposure period for successive trials in order to test the visual-spatial object placement memory span of the child.

#### Number Recall (NR)

In this test, the child must recite a progressively longer list of spoken numbers ranging from one to ten. The numbers was administered in the native language of the children. For NR, the progressively longer sequences of numbers and the response of the child are spoken, so this task depends on auditory memory span. In pre-testing the K-ABC with the 30 elite school 1^st^ grade children in our sample, the auditory span was similar irrespective of whether we presented the numbers in Luganda or in English (*r* (29) = 0.83, *p*<.01). This is despite the differing lengths of phonemes for the same number in Luganda or in English. From this, we concluded that their auditory WM span for this task is mediated by the auditory/phonological loop domain.

#### Word Order (WO)

In this test, the examiner spoke a list of words from a limited set of familiar objects (e.g., cup, house, ball, tree, dog, and star) in the child's local language. The child then pointed to silhouette drawings of those objects in the correct order, when the array of objects is presented immediately after the words are spoken. The spoken list of objects to which the child must point becomes progressively longer with each successive subtest item.

If the child progresses far enough through the subtest, a five second color naming interference task is then introduced between the time the words are recited and when the array of silhouettes is presented so that the child can point to the visual objects for those spoken words in the correct order. The presentation of the words is spoken and must be remembered through auditory means. The response involves pointing to the visual representations for those words. Word Order, therefore, is considered to be a verbal memory test with both auditory and visual (cross-modal) WM components. In our present analysis, the comparative strength of HM (visual-motor), SM (visual-spatial), and NR (auditory) in predicting WO (verbal) WM indicates the extent to which the auditory-phonological loop is dominant for a given group of children.

### Procedure for K-ABC Assessment

Following pilot testing which included translation and back translation of the instructions and items, the K-ABC was administered to the children in the principal local language for that region by native speakers: Wolof for the Senegalese children and Luganda for the Ugandan children. The Senegalese children were all assessed by the same group of three Senegalese adults trained by one of the authors (MJB). These consisted of a Senegalese woman university graduate who led the assessment team along with a man and woman who were secondary school graduates and worked as research assistants for the West African Research Center office in Dakar.

The Ugandan children for the malaria study groups and the 1^st^ grade children at the elite school were assessed by one of the authors (PB) and his research associate (a woman university graduate) trained by MJB [12]. Ugandan children serving as community controls for the HIV study were assessed by a Ugandan university graduate trained by MJB [14].

#### Statistical analysis

The linear regression command of SPSS 17.0 Base Package was used to enter the following predictor variables: age, school grade level, gender, weight in proportion to height, hand movements (visual-motor WM span), spatial memory (visual-spatial WM span), and number recall (auditory-phonological WM span). The predicted variable was word order (verbal WM span). This analysis was completed for the younger age group only (<8.5 yrs) and older age group only (>8.5 yrs) for each culture separately (Uganda, Senegal). For each of these multiple regression analyses by age group and culture, the unstandardized beta coefficient (effect size) and corresponding significance *P* value for each predictor was noted.

## Results

### Comparison of Ugandan and Senegalese Children

The Ugandan children were almost 2 yrs younger than the Senegalese children on average (7.7 yrs versus 9.5 yrs of age), even though the Ugandan children averaged about a year more of schooling (2.7 yrs versus 1.6 yrs of school) (Table 1). The Senegalese children were significantly lower than the Ugandan children on standardized scores from Epi Info CDC2000 norms for weight for age and for body mass index. The two groups were equivalent on height for age standardized scores and proportion of females in the sample (Table 1).

**Table 1:** For age, education, gender, and physical development measures, the means (M) and standard deviations (*SD*), Student *t* test values and significance level probability values (*P*) for Uganda versus Senegal groups.

Measures	Uganda		Senegal		Between-Group Comparison	
	(N = 211)		(N = 76)			
	*M*	*SD*	*M*	*SD*	*t*	*P*
Age (yrs)	7.7	2.2	9.5	2.6	5.96	<0.001
Years of School	2.7	2.0	1.6	1.7	4.03	<0.001
Percent Female N (%)	112 (53%)		35 (46%)			0.294^a^
Weight for Age *Z*	−0.99	1.1	−2.01	2.8	4.46	<0.001
Height for Age *Z*	−0.88	1.2	−0.87	1.8	0.01	0.994
Body Mass Index *Z*	−0.73	1.3	−2.33	3.2	−5.90	<0.001

a Chi Square test used for statistical *P* value.

Physical development *Z* scores based on CDC2000 norms (Epi Info).

When comparing the Senegalese and Ugandan groups on K-ABC memory subtest performance, the only significant difference was for Hand Movements. The two groups did not differ significantly on Spatial Memory, Number Recall, or Hand Movements (Table 2). An analysis of covariance (ANCOVA) was used for these between-culture comparisons using age as the covariate in adjusting the group means and standard errors.

**Table 2:** Kaufman Assessment Battery for Children (KABC) memory subtest raw score unadjusted mean (*M*) and standard deviation (*SD*) values are presented for the Uganda and Senegal groups.

	Unadjusted *M* (*SD*)		Adjusted *M* (*SE*)		ANCOVA Analysis Significance Test	
KABC Memory Test	Senegal	Uganda	Senegal	Uganda	*P*	Effect Size
Hand Movements	9.4 (3.4)	9.0 (3.5)	8.4 (.36)	9.3 (.18)	0.03	0.91
Spatial Memory	9.7 (4.0)	8.1 (4.5)	8.2 (.45)	8.5 (.22)	0.58	0.28
Number Recall	9.5 (2.6)	8.9 (2.8)	8.8 (.30)	9.1 (.15)	0.31	0.35
Word Order	9.7 (4.1)	8.1 (3.9)	8.6 (.42)	8.4 (.21)	0.69	0.19

Also presented are the adjusted Mean (*M*) and standard error (*SE*) values for an ANCOVA analysis with age as the covariate. The significance probability (*P*) value and corresponding effect size for the ANCOVA between-group comparison are presented.

### Multiple Regression Analysis for Ugandan and Senegalese Age Groups

For the Ugandan younger age group (<8.5 yrs), Word Order was significantly predicted by Hand Movements (*P* = 0.006), Spatial Memory (*P* = 0.044), and Number Recall (*P*<.001) (Table 3). For the Ugandan older age group (>8.5 yrs), Word Order was significantly predicted by Hand Movements (*P* = 0.032) and Number Recall (*P*<0.001). For both age groups, the most significant predictor of Word Order (verbal WM) was Number Recall (auditory-phonological WM), with the significance of Hand Movements (visual-motor WM) and Spatial Memory (visual-spatial WM) much weaker for the older age group compared to the younger age group.

**Table 3:** Linear multiple regression unstandardized coefficient (*b*) and statistical *P* values for KABC Hand Movement, Spatial Memory, Number Recall, Years of Schooling, and weight in proportion to height as the predictor variables; and KABC Word Order as the dependent variable.

KABC Memory Test	Uganda		Uganda		Senegal		Senegal	
	<8.5 yrs		>8.5 yrs		<8.5 yrs		>8.5 yrs	
	N = 119		N = 92		N = 34		N = 42	
	*b*	*P*	*b*	*P*	*b*	*P*	*b*	*P*
Hand Movement	0.27	**0.006**	0.25	**0.03**	−0.03	0.86	0.19	0.39
Spatial Memory	0.12	**0.04**	0.09	0.49	0.55	**0.01**	0.46	**0.03**
Number Recall	0.44	**<0.001**	0.57	**<0.001**	0.42	0.06	0.19	0.48
Age in Years	0.14	0.67	0.28	0.41	−0.46	0.42	−0.08	0.80
Gender	−0.17	0.72	0.49	0.45	0.73	0.47	1.34	0.27
Years School	−0.12	0.96	0.13	0.63	0.54	0.18	0.20	0.55
Weight/Height	−7.16	0.51	3.00	0.82	−12.93	0.42	24.10	0.16

Both younger (<8.5 yrs) and older (>8.5 yrs) age groups are presented for the Uganda and Senegal samples. *P* values for significant coefficients are in bold.

For the Senegal younger age group (<8.5 yrs), Word Order was significantly predicted by only by Spatial Memory (*P* = 0.011) (Table 3). For the Senegal older age group (>8.5 yrs), Word Order was again only significantly predicted by Spatial Memory (*P* = 0.033). For both age groups, the most significant predictor of Word Order (verbal WM) was Spatial Memory (visual-spatial WM). Number Recall (auditory-phonological WM) did not significantly predict Word Order for either age group.

## Discussion

### Overview of Multiple Regression Findings

Both the younger (<8.5 yrs) and older (>8.5 yrs) Ugandan children had auditory memory span (Number Recall) that was strongly predictive of Word Order performance. For both the younger and older groups of Senegalese children, only visual WM span (Spatial Memory) was strongly predictive of Word Order; Number Recall was not significantly predictive of Word Order in either age group.

It is possible that greater literacy from more schooling for the Ugandan age groups mediated their greater degree of interdependence between auditory and verbal WM. This is supported by the fact that if age is dropped from the multiple regression analysis depicted in Table 3, grade level becomes significantly predictive of Word Order for the Ugandan children (*P*<0.001), but not for the Senegalese children (*P* = 0.32). Uganda maintains a very strong universal education policy, and almost all of our study children were from an urban environment and enrolled in school at the time of testing. School enrollment in Senegal was much less consistent, especially for the rural samples of children. This would suggest that education and subsequent literacy is the critical factor in the extent to which visual and auditory working memory systems become less separate and distinct from one another, allowing auditory-phonological WM to become dominant in verbal WM.

Our findings in the present cross-cultural WM analysis support those of Conant et al., who observed in their cross-cultural comparisons that stronger education seemed to enhance the dominance of the phonological-auditory processing loop for WM. This tendency was apparent in earlier studies by Conant of Lao and American children, and supports the suggestion that as literacy is acquired by children, the auditory/phonological memory system becomes more dominant in cross-modal tasks and perhaps in WM in general.

Unfortunately, we did not specifically test degree of literacy in our study children, so we cannot confirm this hypothesis in the present analysis. This should be a priority in subsequent investigations. It would be especially interesting to evaluate the correlation between visual and auditory working memory before and after children at various age levels become literate.

### Relevance of Conant et al. Findings with American, Congolese, and Lao Children

Conant suggested that the stronger degree of literacy training for the American children in school, when compared to that of the Congolese children, may have mediated a developmental shift towards a greater reliance on verbal/semantic processing of memory for sequential lists of either auditory information (number recall, word order), or visual/motor information (sequences of hand movements).

Conant et al. (1999) also conceded the possibility that the overall favorability of the developmental milieu (e.g., nutritional resources and health status) for children at risk from pervasive poverty might modify brain development so as to delay a natural progression in working memory towards a greater propensity for and reliance upon verbal/semantic processes. For such children, this might be the case irrespective of level of schooling or literacy training *per se*, and may have been the case for her sample of children from DR Congo.

Nutritional status was poorer for our Senegal compared to the Ugandan children (Table 1), but educational status was also lower for the Senegal sample. In our multiple regression analysis, we included level of schooling and nutritional status (weight in proportion to height). Number Recall (auditory-phonological WM) still emerged as the most significant predictor of verbal WM in the Ugandan age groups, while Spatial Memory (visual-spatial WM) still emerged as the most significant predictor of verbal WM for both Senegal age groups.

However, we need to evaluate our WM regression model in a sample of Ugandan children from a more impoverished nutritional and educational background and include health indicators (e.g., CBC blood count profiles for anemia and infection). The expanded scope of such an analysis would allow us to more effectively test the extent to which the greater propensity for and reliance upon verbal/semantic processes is dependent on literacy, or dependent on health and integrity of brain/behavior development.

### Mechanisms for the Dynamic Shift in WM System Dominance

Different theories have been given to explain why younger children rely more on visual cues and older children on verbal. Hitch et al., (1988) conducted five experiments to see how children processed visual tasks. The young children used the visual-spatial system of working memory to remember sequences of drawings while older children were more likely to rely more on the phonological loop to store labels for the drawings. The authors proposed that there is a passive storage of visual information in children and adults. In children, this storage has a smaller capacity than with adults, so they have to use other ways of remembering. They are more likely to store things by the ways they look, focusing on such features as shape, color, and placement [15].

Hitch et al., (1989) did a further study to explore whether older children have a component of visual memory coding that they can use even if they primarily rely on phonological coding. The authors proposed that the visual coding may be masked by the phonological component. The experiment did memory recall with groups of five year olds and eleven year olds. With the five year olds, there was evidence that confirmed the previous findings by Hitch et al. (1988) that children do use some sort of visual storage rather than phonological storage.

With the older children, Hitch and colleagues used an articulatory suppression task in order to specifically see the visual working memory elements. When this was done, the older children were similar to the five year olds in that the older children used a visual storage strategy to recall the information [16]. Hitch and colleagues concluded that older children still use the visual component of WM, but it is usually masked by the more dominant phonological memory. Visual coding WM strategies favored by younger children do not disappear with age. They simply are not needed as much as phonological coding capacity and efficiency increase with age.

### Conclusions

Conant et al. (1999, 2003) found that visual-spatial and auditory-verbal WM were independent from one another in younger Congolese, Lao and American children. In older children, the two memory systems were significantly correlated with one another for American and Lao, but not for the Congolese children.

The developmental change in the strength of relationship between the two WM systems with Lao and American children observed by Conant et al. (2004) was hypothesized to be mediated partly by literacy training, which enhances the dominance of the phonological-auditory processing loop in WM. It is possible that greater literacy from more schooling for the Ugandan age groups mediated their greater degree of interdependence between auditory and verbal WM. Thus, our findings seem to support those of Conant et al., who observed in their cross-cultural comparisons that stronger education seemed to enhance the dominance of the phonological-auditory processing loop for WM.

However, a more rigorous test of this model needs to include a more thorough array of health and nutrition indicators for children in impoverished environments. These would indicate those children at significant risk for diminished or delayed brain/behavior development which might preclude the manner in which literacy tends to facilitate WM visual/auditory semantic differential progression. Therefore, we still leave open the possibility that beyond the role of education and literacy *per se*, impoverished children are at risk for delayed neurocognitive development because of compromised brain/behavior development [17], [18].

Such brain/behavior development risk factors may better explain why even when schooling is available, severely impoverished children may rely on visual-spatial WM for a longer period or be delayed in their developmental transition to auditory-phonological WM as the dominant system. This also has important implications for the for the types of interventions needed to best enhance the neurocognitive trajectory of these children [19].

## References

[1] Conant L, Fastenau P, Giordani B, Boivin M, Chounramany C (2003). Environmental Influences on primary memory development: a cross-cultural study of memory span in Lao and American children.. Journal of Clinical and Experimental Neuropsychology.

[2] Baddeley AD (2001). Is working memory still working?. Am Psychol.

[3] Gathercole S, Pickering S, Ambridge B, Wearing H (2004). The structure of working memory from 4 to 15 years of age.. Developmental Psychology.

[4] Acredolo LP, Pick HL, Olsen MG (1975). Environmental differentiation and familiarity as determinants of children's memory for spatial location.. Developmental Psychology.

[5] Wellman H, Ritter A, Flavell JH (1975). Deliberate memory behavior in the delay reactions of very young children.. Developmental Psychology.

[6] Appel LF, Cooper RG, McCarrell N, Sims-Knight J, Yussen SR (1972). The development of the distinction between perceiving and memorizing.. Child Development.

[7] Kaufman AS, Kaufman NL (1983). Kaufman Assessment Battery for Children: Administration and Scoring manual.. Circle Pines.

[8] Conant LL, Fastenau PS, Giordani BJ, Boivin MJ, Opel B (1999). Modality specificity of memory span tasks among Zairian children: A developmental perspective.. J Clin Exp Neuropsychol.

[9] Vygotsky LS (1978). Mind in Society: The Development of Higher Psychological Processes. Cambridge, MA: Harvard University Press..

[10] Boivin MJ (2002). Effects of early cerebral malaria on cognitive ability in Senegalese children.. J Dev Behav Pediatr.

[11] Bangirana P, John CC, Idro R, Opoka RO, Byarugaba J (2009). Socioeconomic predictors of cognition in Ugandan children: Implications for community based interventions.. PLoS ONE.

[12] Boivin MJ, Bangirana P, Byarugaba J, Opoka RO, Idro R (2007). Cognitive impairment after cerebral malaria in children: a prospective study.. Pediatrics.

[13] John CC, Bangirana P, Byarugaba J, Opoka RO, Idro R (2008). Cerebral malaria in children is associated with long-term cognitive impairment.. Pediatrics.

[14] Bagenda D, Nassali A, Kalyesubula I, Sherman B, Drotar D (2006). Health, neurologic, and cognitive status of HIV-infected, long-surviving, and antiretroviral-naive Ugandan children.. Pediatrics.

[15] Hitch G, Halliday S, Schaafstal A, Schraagen J (1988). Visual working memory in young children.. Memory and Cognition.

[16] Hitch G, Woodin M, Baker S (1989). Visual and phonological components of working memory in children.. Memory and Cognition.

[17] Grantham-McGregor S, Cheung YB, Cueto S, Glewwe P, Richter L (2007). Developmental potential in the first 5 years for children in developing countries.. Lancet.

[18] Walker SP, Wachs TD, Gardner JM, Lozoff B, Wasserman GA (2007). Child development: risk factors for adverse outcomes in developing countries.. Lancet.

[19] Engle PL, Black MM, Behrman JR, Cabral de Mello M, Gertler PJ (2007). Strategies to avoid the loss of developmental potential in more than 200 million children in the developing world.. Lancet.

