# Correction: The Relationship between Visual-Spatial and Auditory-Verbal Working Memory Span in Senegalese and Ugandan Children

**DOI:** 10.1371/annotation/b9e2fb60-c9b6-4d00-9f09-69394cf2da10

**Published:** 2010-02-24

**Authors:** Michael J. Boivin, Paul Bangirana, Rebecca C. Shaffer

The third author's name and affiliation have changed. The correct name is Rebecca C. Shaffer. The correct affiliation is: Chestnut Hill College, Philadelphia, Pennsylvania, United States of America. The correct citation is: Boivin MJ, Bangirana P, Shaffer RC (2010) The Relationship between Visual-Spatial and Auditory-Verbal Working Memory Span in Senegalese and Ugandan Children. PLoS ONE 5(1): e8914. doi:10.1371/journal.pone.0008914

